# Transplanting Intact Donor Tissue Enhances Dopamine Cell Survival and the Predictability of Motor Improvements in a Rat Model of Parkinson’s Disease

**DOI:** 10.1371/journal.pone.0047169

**Published:** 2012-10-09

**Authors:** Rosemary A. Fricker, Jan Herman Kuiper, Monte A. Gates

**Affiliations:** 1 Keele University, School of Life Sciences, Institute for Science and Technology in Medicine, Keele, United Kingdom; 2 Institute for Science and Technology in Medicine, Keele University, Keele, United Kingdom; 3 RJAH Orthopaedic Hospital, Oswestry, United Kingdom; The Mental Health Research Institute of Victoria, The University of Melbourne, Australia

## Abstract

Primary cell transplantation is currently the gold standard for cell replacement in Parkinson’s disease. However, the number of donors needed to treat a single patient is high, and the functional outcome is sometimes variable. The present work explores the possibility of enhancing the viability and/or functionality of small amounts of ventral mesencephalic (VM) donor tissue by reducing its perturbation during preparation and implantation. Briefly, unilaterally lesioned rats received either: (1) an intact piece of half an embryonic day 13 (E13) rat VM; (2) dissociated cells from half an E13 rat VM; or (3) no transplant. D-amphetamine- induced rotations revealed that animals receiving pieces of VM tissue or dissociated cells showed significant improvement in ipsilateral rotation 4 weeks post transplantation. By 6 weeks post transplantation, animals receiving pieces of VM tissue showed a trend for further improvement, while those receiving dissociated cells remained at their 4 week scores. Postmortem cell counts showed that the number of dopaminergic neurons in dissociated cell transplants was significantly lower than that surviving in transplants of intact tissue. When assessing the correlation between the number of dopamine cells in each transplant, and the improvement in rotation bias in experimental animals, it was shown that transplants of whole pieces of VM tissue offered greater predictability of graft function based on their dopamine cell content. Such results suggest that maintaining the integrity of VM tissue during implantation improves dopamine cell content, and that the dopamine cell content of whole tissue grafts offers a more predictable outcome of graft function in an animal model of Parkinson’s disease.

## Introduction

The development of primary cell transplantation as a therapeutic treatment for Parkinson’s disease (PD) has evolved a great deal since early studies provided proof for its potential efficacy in treating the disease clinically [1], [2], [3], [4], [5]. Though hopes remain for the generation of an unlimited source of dopamine neurons from a stem cell line [6], [7], primary cell transplantation currently remains the gold standard for a cell replacement strategy in late stages of the disease, when the efficacy of pharmacological therapy wanes [2], [3], [8]. Though the outcome of primary cell transplants appears largely positive [9], there is still some degree of inconsistency [10] as well as practical considerations that make it difficult for the strategy to be more widely used [4], [11]. Among practical concerns is the difficulty in obtaining a suitable number of donors (at a given time) for grafting into a single patient. Past studies, in fact, have indicated that anywhere between 1 to 8 embryonic donors might be needed to treat a single patient via primary cell transplantation [12], each of which should be within a narrow time window of gestation. A current review of the dopamine cell replacement strategy also highlights the fact that the viability and functionality of transplanted cells can be compromised, and that recipients can suffer dyskinesias after transplantation [10]. If primary cell transplantation is to become more practical for widespread use, the yield of tissue from each donor will have to be maximised, and the transplanted tissue will have to provide more consistent outcomes.

The current use of such a large number of precisely aged donors is due mainly to the fact that a vast number of donor neurons are destroyed during the preparation of cell suspensions for transplantation [4]. Seminal work by Brundin and colleagues [13], in fact, has shown that only between 3–20% of donor cells survive standard preparation and transplantation procedures [14]. This, it is thought, is mostly due to a cascade of destructive events brought on by oxidative stress, some of which can be combated to increase the yield and viability of cells from a single donor [13]. Past studies have shown that the yield of dopamine neurons from primary sources can be improved by incubating tissue in “hibernation” factors, and/or placing tissue in a mild hypothermic state during their preparation [15], [16]. More recent work (in rats) has shown that the yield of primary dopamine neurons can also be enhanced by extracting them at an early gestational age. Studies comparing the final yield of tyrosine hydroxylase positive (TH+) neurons in transplants made from rat donors at either E11, E12, E13 or E14 days of gestation have shown that transplants from embryonic day 12 (E12) donors maintain a greater number of viable dopamine neurons [17], [18], [19]. This seems to indicate that obtaining cells around the developmental stage of peak differentiation of the A9 dopamine neuron group, but before they have established long neuritic processes [20], [21], may greatly improve the yield of cells from each individual donor. This has recently proven true in mice, where the birthdate of the A9 group of dopamine neurons has been directly correlated with both increased donor TH neuronal survival after transplantation, as well as improved innervation of the striatum by dopamine transplants [22].

As the developmental age at which the maximum number of donor neurons may be harvested has reduced, so too has the size of VM tissue dissected for primary cell transplantation in the laboratory rodent model of PD. The size of the tissue in rats is now sufficiently small that whole piece of one side of the VM of an appropriate aged donor (E12 or E13) can be readily loaded (intact) into a standard (26-gauge) implantation needle, and subsequently grafted. The importance of this development is that it now allows for the implantation of intact pieces of donor VM tissue, circumventing any untoward effects that cell dissociation (or tissue distortion) may have on neuronal survival or function, during or after transplantation procedures. The present work has sought to draw on this development by testing whether fully maintaining the integrity of a dissected piece of VM tissue during implantation improves the viability and/or functionality of transplanted mesenecephalic dopamine neurons in comparison to the single cell suspension method. This was done by dissecting VM tissue from E13 rats, and bisecting the tissue evenly. One piece remained intact (unperturbed) during implantation, and the other half was prepared via traditional dissociation methods before transplantation. A comparison was made of not only the level of dopamine cell survival in the grafts, but also the reversal of rotation bias in relation to the level of dopamine neuron survival in grafts. It is hoped that this work will shed some light on the impact that cell dissociation may have on dopamine cell survival and functioning in cell replacement for PD, and provide some insight into the potential for placing small pieces of intact tissue into the host brain in a way that has a positive therapeutic outcome.

## Materials and Methods

All *in vivo* procedures were performed in conjunction with the Animals (Scientific procedures) Act 1986, including approval from the animal care and use committee at Keele University. Experimental animals used for the study consisted of adult female Sprague Dawley rats (weighing ∼230 g), housed in a 12/12 h light-dark environment while given free access to food and water throughout the study.

### Unilateral 6-OHDA Lesions

Rats were anesthetised with gaseous isoflurane and placed in a stereotaxic frame with the incisor bar set at −4.5 mm. After a midline incision, a small hole was drilled in the skull 4.0 mm anterior to the bregma suture, and 1.3 mm lateral (right side only) to the midline. A fine glass capillary, filled with a 30 mM 6-OHDA (Sigma)/0.03% ascorbic acid solution, was lowered 7.0 mm ventral to the cortical dura, and 3 µl of the solution injected (using a Nanoject; Drummond) into the right medial forebrain bundle over a 3 minute period of time. The capillary was left in place for an additional 2 minutes, then subsequently removed and the animal sutured and placed in a warmed cage for recovery.

### Amphetamine Rotations

To assess the effectiveness of unilateral lesioning of the nigro-striatal circuit, and to establish baseline scores for pre-transplantation rotational bias, animals were assessed using d-amphetamine treatment. Briefly, at 2 weeks post-lesioning, animals received a 5 mg/kg i.p. injection of d-amphetamine sulphate (Sigma). Full rotations, both left and right, were scored over a 90 minute period of time, and net ipsilateral turns per minute calculated. Four weeks post-lesioning a second rotational analysis was similarly performed and the average of the two pre-transplantation rotational scores for each animal used to assign animals evenly in one of three groups: (1) animals that would receive transplants of an intact piece ventral mesencephalic (VM) tissue from one side of an E13 rat donor brain (n = 6); (2) animals receiving transplants of dissociated VM cells from the other half of the E13 donor brain (n = 8); and (3) control animals receiving no transplants (n = 8). Subsequently, d-amphetamine induced rotations were carried out both 4 and 6 weeks post transplantation, and the rotation scores of all animals (transplant and control) were tabulated as above, with net turns per minute calculated.

### Dissection and Transplantation

Two weeks after baseline rotational analysis, animals in the two experimental groups received transplants of primary cells or tissue pieces from the embryonic day 13 (E13) rat ventral mesencephalon (VM). Briefly, donor animals were obtained from time mated female rats, with the day of plugging noted as embryonic day 0 (E0). The developmental age was further confirmed via crown-rump (C-R) measures at the time of dissection [17], [18], with the average C-R length for donor animals used being between 7.0–7.5 mm. The mesencephalon was cut free of whole embryos in ice cold dissection media (95% DMEM, 2% or 30% Glucose, 1.6% of a 7.5% NaHCO_3_ solution, and 0.5% of 1 M HEPES buffer), and further trimmed to isolate the ventral portion. It is important to note that after removing the outer meningeal tissue, the VM was bisected evenly along the midline and placed in one of two Eppendorf tubes filled with dissection media. One half of the VM was placed in a tube marked for “dissociation”, the other was placed, intact, in a separate tube marked “intact” for direct implantation. Tissue marked for dissociation was washed in dissection media containing 0.05% DNase and incubated for 20 minutes in 0.1% trypsin at 37°C. Cells were dissociated via trituration through a Gilson 200 µl pipette tip, and spun on a benchtop centrifuge for 5 minutes at 1200 rpm. The cells were subsequently resuspended in culture media (86% Neurobasal, 10% fetal calf serum, 1% B27, 1% Pen/Strep/Fun, 1.5% of 30% Glucose, and 0.5% Glutamine) at a final concentration of 3 µl per half VM dissected. Pieces of tissue designated for use “intact” were rinsed in DNase-containing buffer and placed in a 24 well plate containing culture media (the same as that used for the cell suspensions).

For transplantation of the cell suspension, 3 µl of dissociated cells were drawn up into a fine glass capillary fixed to a Nanojector injection system (Drummond), and the Nanojector secured onto a stereotaxic frame. For transplantation of whole pieces of donor tissue, an intact half VM piece from an E13 donor was gently drawn up (free floating) into a 2 µl Hamilton syringe with a fixed needle (outer diameter of 480 µm) using a high power dissecting microscope to observe the fit of the tissue into the needle. The size of the piece of tissue was made so that it did not exceed the inside diameter of the needle, and that the tissue piece was not noticeably deformed during the loading procedure (virtually free floating inside the need shaft). To transplant either intact pieces of half an E13 VM or the dissociated cell equivalent, 6-OHDA lesioned animals were anesthetised with isofluorane, and placed in a stereotaxic frame with the nose bar set at −2.3 mm. After a midline incision, a small hole was drilled +0.6 mm rostral to the Bregma suture, and 3.0 mm lateral (right) to the midline. Subsequently, either the Hamilton needle containing an intact piece of half VM, or the glass capillary containing the dissociated cell equivalent, was lowered 4.5 mm deep below the cortical dura to transplant (either) the tissue or cells (respectively) into the denervated striatum. The needle or capillary was left *in situ* for 2 minutes before gentle removal, and the animal sutured and placed in a warm cage for recovery.

### Histology

One week after final post-transplantation rotation analysis, animals were given an overdose of pentobarbitone anesthetic and cardiac perfused with a 1.5% solution of paraformaldehyde (PFA) in Tris-buffered saline (TBS). The brain was removed and placed in PFA overnight. The following day, specimens were transferred to a 30% sucrose solution in TBS and allowed to fully sink in the solution at room temperature (RT). Subsequently, specimens were placed on a sliding, freezing microtome and 40 µm coronal sections cut through the entire striatum. Sections were placed in 24-well plates containing TBS with sodium azide, with 1 in 6 sections being taken for immunohistochemistry using tyrosine hydroxylase (TH) antibodies. For immunohistochemistry, sections were rinsed 3× times in TBS, and subsequently incubated in a blocking solution containing 3% goat serum/TBS for 1 hour at RT. The blocking solution was subsequently replaced with a 1∶1000 dilution of mouse anti-TH antibody (Sigma) in TBS containing 1% goat serum, and left to incubate overnight at RT. The following day, sections were rinsed 3× in TBS followed by a 2 hour incubation in a 1∶200 dilution of a biotinylated goat anti-mouse secondary antibody (DAKO) in 1% goat serum/TBS. Subsequently, sections were rinsed 3× in TBS, followed by a 1 hour incubation in a solution containing a 0.5% dilution of the avidin-biotin complex (Vector). After rinsing 3× in TBS, sections were “reacted” for 5 minutes in 0.5 mg/ml of diaminobenzadine (Sigma)/TBS containing 1.2 ul/ml of 30% H_2_O_2_. Dopamine neuron numbers were determined as a total TH+ cell counts in 1 in 6 sections were done blinded (using a 10× power on a light microscope), and the Ambercrombie correction formulae applied to determine the total number of TH+ cells per graft in each animal [23].

### Statistical Analysis

All data were checked for normality and outlying values using Q-Q plots. If suggested by the plots, the presence of outlying values was confirmed using Grubbs’ test. Homoscedasticity of rotational bias was assessed using Levene’s test. Rotational bias was normally distributed and homoscedastic. Mean rotational bias within each group at pre-, and 4 and 6 weeks post- transplantation was compared using paired t-tests. Mean rotational bias between the 3 groups at pre-, and 4 and 6 weeks post- transplantation was compared using one-way ANOVA followed by Tukey’s post-hoc test. TH+ cell counts in both experimental groups had one outlying value. Mean cell counts were therefore compared using a method robust to outlying values, the bootstrapped t-test based on 20% trimming [24]. The relation between amphetamine rotation and cell counts in the two experimental groups was investigated using Pearson’s correlation coefficient, and its significance assessed using a t-test. Data is presented as means ±SEM. Means of cell counts were calculated as 20% trimmed means, and their SEM calculated from the 20% Winsorized standard deviation [24]. Statistical analyses were performed using Excel 2003 (Microsoft, Redmond, WA) and R vs. 2.15.1 (R Foundation for Statistical Computing, Vienna, Austria) using the packages “outliers” and “WRS”. All t-tests were two-tailed, and a p-value below 0.05 was assumed to denote significance.

## Results

### Amphetamine Rotations

**Figure 1:**
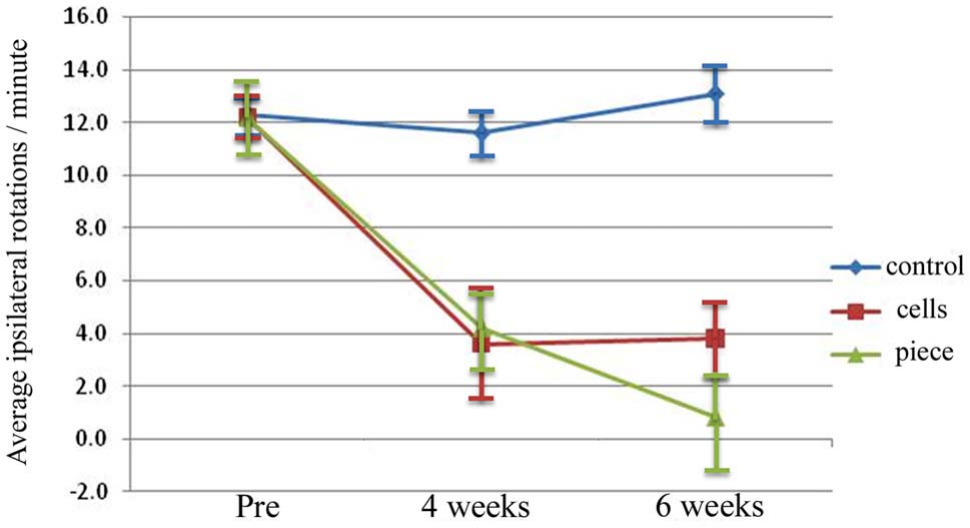
Dissociated cells and piece of VM tissue improve rotation bias in unilaterally 6-OHDA lesioned rats. Net ipsilateral rotation scores of animals pre-transplantation (Pre), and 4 and 6 weeks after transplantation of dissociated cells from, or an intact piece of, half an E13 VM versus controls (lesion alone). Data are shown as group mean rotations of control (diamonds), cell transplanted (squares), and whole tissue transplanted (triangle) animals, with bars above and below each data point indicating the ± SEM. Though both groups of animals receiving either transplants of dissociated VM cells (squares) or intact pieces of VM tissue (triangles) recovered some rotational symmetry, animals receiving transplants of pieces of VM tissue continued to improve after 4 weeks post-transplantation to nearly zero rotations per minute by 6 weeks post-transplantation.

At 2 and 4 weeks following 6-OHDA lesioning all animals in the study displayed an ipsilateral (to the lesion) rotation bias after d-amphetamine administration. Mean rotation scores ranged from 7.3 turns per minute (tpm: over a 90 minute period of time), to 17.3 tpm. Animals were then placed in groups so that the average rotation scores of the 3 groups before transplantation were: 12.2 tpm (+/−1.42) for animals to be transplanted with pieces of tissue; 12.2 tpm (+/−0.84) for those to be transplanted with dissociated cells; and 12.3 tpm (+/−0.78) for control animals (Figure 1). Four weeks following cell replacement, animals that had received transplants of intact pieces of VM tissue showed a significant reduction in average ipsilateral rotation scores (to 4.2 tpm, +/−1.11; p<0.001 compared to pre-implantation) as did those receiving transplants of dissociated VM cells (3.6 tpm, +/−2.14; p<0.001 compared to pre-implantation). This was in contrast to control animals which maintained a high ipsilateral rotation bias (11.6 tpm, +/−0.85). By 6 weeks post-transplantation, animals that had received intact pieces of VM tissue showed some further improvement in rotation bias, with ipsilateral rotation scores of 0.8 tpm, +/−1.9, while animals that had received dissociated cells from half a VM maintained a similar rotation bias to their 4 week levels (3.8 tpm, +/−1.45). At 4 and 6 weeks, rotational bias of both experimental groups differed significantly from the control group (p<0.001 for each comparison). However, even at 6 weeks no significant difference between the two experimental groups was found (p = 0.344).

### Histology

**Figure 2:**
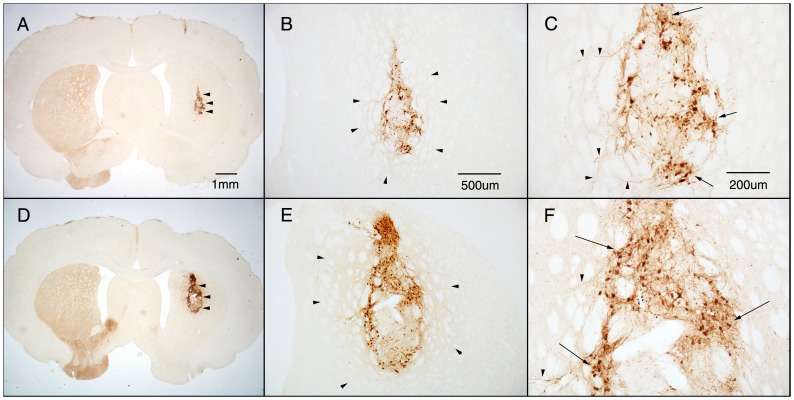
Dissociated cell and whole tissue transplants contain TH+ cells projecting neuritis into the host striatum. Tyrosine hydroxylase (TH) staining of coronal sections through transplants of dissociated cells (A–C), or intact pieces of tissue (D–F) from one half of an E13 VM. (A and D) Transplants (arrowheads) were placed centrally in the 6-OHDA denervated striatum of lesion rats. (B and E) Numerous TH+ cell bodies could be seen situated within grafts of either dissociated cells (B) and whole tissue (E), with a halo (arrow heads) of diffuse TH+ cell fibers distributed throughout much of the surrounding host striatum. (C and F) High power images show varied TH+ cell profiles (arrows) throughout both types of grafts, and large, tapering TH+ processes innervating the surrounding host parenchyma (arrow heads). Scale bar for D shown in A, for E shown in B, and for F shown in C.

**Figure 3:**
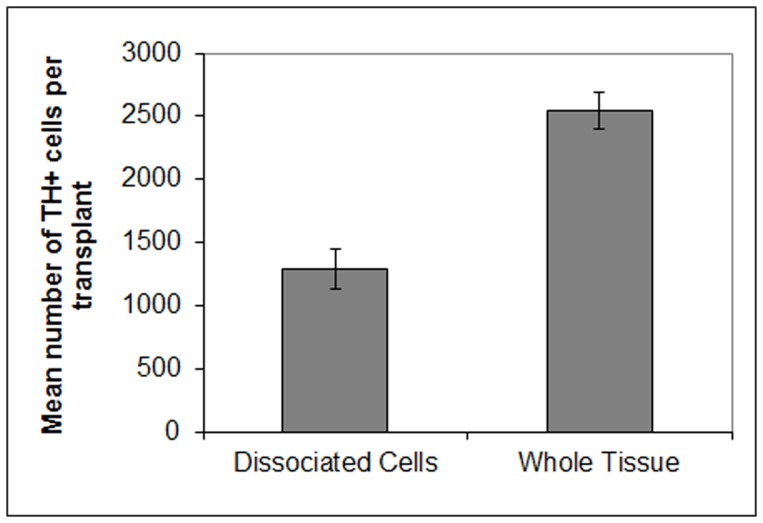
The number of TH+ cells was greater in animals receiving pieces of E13 VM tissue. After Ambercrombie correction, the total number of TH+ cells in animals receiving dissociated cells from the E13 VM averaged 1290 (+/−159) per transplant. In comparison, grafts of pieces of VM tissue contained significantly greater numbers of TH+ cells 2543 (+/−142; p = 0.003) 6 weeks after transplantation.

Immunohistochemistry, using an antibody to TH (Figure 2), revealed placement of the transplants in the central core of the denervated striatum (Figure 2A, D). Though the size of TH+ region of grafts of pieces of tissue appeared generally larger than dissociated cell transplants, variability was notable in both treatments. In both intact VM pieces and dissociated cell transplants, TH+ staining could be seen distributed throughout much of the border between the transplant and the host, with a fine halo of TH+ fiber staining in the surrounding host striatal tissue (Figure 2B, E). Dopaminergic cells within each transplant showed dense cytoplasmic staining, with a range of process outgrowth from short single processes to branching dendritic arbours (Figure 2C, F). Counts of TH+ cells in transplants of either pieces of VM tissue or dissociated VM cells showed a significant difference in the number of surviving dopamine neurons when comparing the two transplanted groups (p = 0.003; Figure 3). Whereas TH+ cell counts in animals receiving dissociated E13 VM cells showed an average of 1290 (+/−159) dopaminergic neurons per transplant (after Abercrombie correction), grafts of pieces of VM tissue contained 2543 (+/−142) TH+ neurons on average.

### Rotations and Dopamine Neuron Numbers

**Figure 4:**
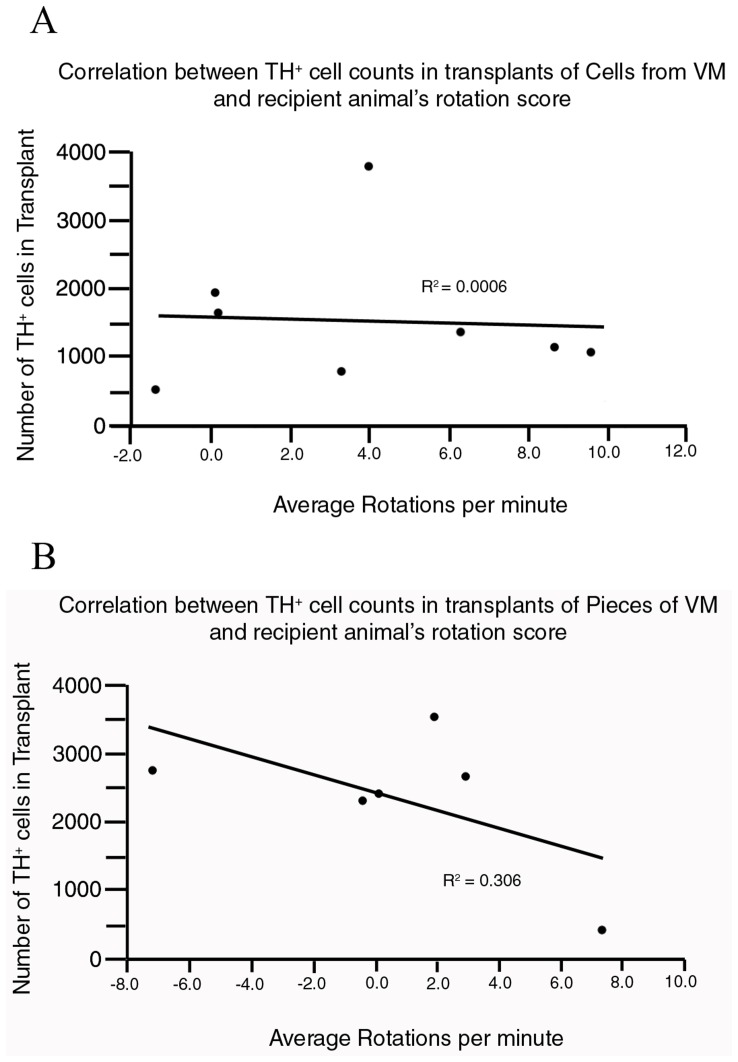
Animals receiving whole tissue transplants display a correlation between dopamine cell numbers and rotation bias. Each data point represents the number of TH+ cells from a single host (y-axis) versus their rotation score (x-axis). Note (A) how there is no correlation between the number of TH+ cells in dissociated cells transplants and their rotation scores (r^2^ = 0.0006), while there is (B) a significant inverse correlation between the number of TH+ cells and ipsilateral rotation in animals receiving transplants of intact pieces of VM tissue (r^2^ = 0.306; p = 0.04).

Further analysis, looking at the relationship between the number of dopamine neurons in grafts and the improvement in rotation scores by animals, revealed an interesting trend (Figure 4). Though there appeared to be virtually no correlation between the number of TH+ cells and rotation scores in animals that had received dissociated cell transplants (r^2^-value = 0.0006; p = 0.93; Figure 4A), a high and significant correlation between the amount of improvement in rotation and the number of TH+ cells was found in animals that had received grafts of pieces of tissue (r^2^-value = 0.306; p = 0.04; Figure 4B).

## Discussion

The present study illustrates the potential importance of maintaining the integrity of VM donor tissue during its preparation for, and implantation into, animal models of Parkinson’s disease (PD). Though both dissociated cells and whole pieces of tissue from one half an E13 VM were able to ameliorate rotation bias in 6-OHDA lesioned animals, maintaining tissue integrity throughout implantation procedures offered the possibility to increase the number of donor dopamine neurons surviving transplantation, and proved to be a better predictor of graft function based on dopamine cell content. Future studies should explore further the possibility that the trend of improvement of rotational bias seen in animals receiving transplants of intact VM tissue between 4 and 6 weeks (when animals receiving dissociated VM cells had levelled out) might continue to progress over time. This may give further evidence that less donor tissue is required to bring about a suitable and predictable behavioural (motor) outcome in lesioned animals. Also, it will be important to understand why donor tissue that is maintained intact during implantation procedures offers a more reliable correlation between the number of surviving dopamine neurons in the grafts and the behavioural outcome of the transplants.

Overall, the present study has taken advantage of the emerging trend of using less mature (thereby smaller) tissue as a source of donor cells for transplantation in the rodent model of Parkinson’s disease, by exploring whether implanting intact pieces of ventral mesencephalon (VM) offers any improvement (on cell survival and/or motor behaviour) above standard dissociated cell transplants. In the past, a certain level of comparison has been made between dissociated cell transplants and less disrupted tissue in the rodent model of PD [25]. The seminal works of Clarkson and Freed, in particular, assessed the effects that dissociating primary cells has on donor cell survival by dissecting pieces of E15 rat VM, and comparing single cell suspensions to “strands” of VM tissue made by forcing the dissected tissue through a tapered glass needle [25]. The advantage of producing strands in this way is that they can then be loaded into a standard, 24 g cannula and implanted into the recipient [12], [25]. However, the procedure still involves a certain level of perturbation of the dissected tissue (i.e., to make the strands) and the standard donor animals used at that time were at a developmental stage (i.e., E14/15) when most dopamine neurons in the VM are now known to have established very long axonal processes [26]. It is likely that these axonal projections would have been sheared during the dissection procedure itself, compromising the neurons’ viability despite the method of preparation used (strands versus single cells). In a similar way, work by Barker et al. [27] showed that when preparing suspensions of VM tissue, both the age of the tissue and the tool used for mechanical dissociation affected TH neuron viability. Dissecting older embryos (E15), and using a fine gauge needle rather than flame-polished glass pipette led to lower numbers of surviving TH neurons within *in vitro* cultures. It is likely that axonal damage was increased in both cases.

In the current study, E13 (7–7.5 mm CR length) rat VM tissue was used as a donor source. This stage of development is now known to be at a time when a vast majority of A9 dopamine neurons have fully differentiated, but very few (if any) have extended fibers beyond the dissected VM region [21], [26]. Also, the size of the E13 VM tissue (when dissected into individual halves) readily fits into a standard implantation (26-gauge, 2 µl Hamilton) needle, without the need to distort the tissue in any way. This, then, allows for the loading, without force, of VM dopamine neurons and their leading processes without tissue distortion or shearing of dopamine processes. When putting the number of TH+ cells surviving in grafts of pieces of VM tissue here in context with earlier work, it is notable that tissue transplants here display an average of 2543 (+/−142 SEM) TH+ neurons per animal. This is substantially greater than that seen in work (531+/−113 SEM) using tissue strands [25], suggesting that even slight distortions of VM tissue may be enough to compromise dopamine neuron viability during transplantation procedures. However, there is the additional issue that earlier embryonic tissue was used in the present study, and this may also dramatically increase the yield of TH+ cells in transplants. Here, dissociated E13 VM transplants displayed 1290 (+/−159 SEM) cells per transplant in comparison to that seen when similar studies used E15 animals (117+/−35 SEM, [25]). This highlights the dramatic improvement that can be brought about through the use of embryonic tissue taken at an early stage of dopaminergic neuron development, even if the cells are dissociated. It will perhaps be important in the design of future clinical treatments to consider standardising both the age of donor tissue and the means by which this tissue is prepared and implanted. It is likely that both of these issues can dramatically affect the proportion of dopamine neurons that survive the grafting procedure, and may improve the reliability of function of those transplants.

Behaviourally, the present work shows that half a VM dissected from E13 rats may be implanted, intact, into the 6-OHDA denervated rat striatum where it can produce a near complete alleviation of rotation bias (from an average of 12.2 rotations to 0.85 rotations per minute) 6 weeks after transplantation. When compared to dissociated cell transplants, maintaining tissue integrity provided an increase in the number of surviving TH+ neurons after transplantation (from 1290+/−159 SEM to 2543+/−142 SEM; Figure 3). The more predictable behavioural outcome in animals that received grafts of whole tissue (based on the dopamine neuron content in transplants) also suggests the possibility that maintaining tissue integrity during preparation and implantation may selectively aid the survival of more functional dopamine neurons [22], [31]. This is supported by the correlation shown between the number of TH+ neurons in transplants and improvement of rotation scores (Figure 4). Though there was virtually no correlation between the number of TH+ neurons and the recovery from rotation bias among animals receiving transplants of dissociated VM cells (reiterating the very high variability in behavioural outcomes in recipients of dissociated cell transplants [10], [28], [29]), a significant correlation was found in animals receiving intact tissue transplants. This illustrates that some of the amelioration of rotation bias in animals receiving whole tissue transplants can be accounted for by the fact that the tissue remained intact during implantation procedures. It seems possible, therefore, that grafts of pieces of tissue might provide for greater functionality over dissociated cells through the preservation of A9 dopamine neurons. Past work has shown that the presence of A9 neurons (and not the A10 ventral tegmental area group) is crucial to the connectivity and functioning of grafted VM cells in the host striatum [30], [31]. Also, studies have shown that it is the loss of specifically the A9 group that has the most dramatic effect on rotation bias in animals [32] and that they are essential to replace if rotation bias is to be recovered in lesioned animals. Though ways to selectively preserve the A9 cells in primary cell transplants needs further exploration, what is certain is that it will be crucial clinically to not only consider how to maximise the number of TH+ neurons available for grafting from each donor, but to also increase the ratio of the A9 dopamine group in grafts to reduce variability in outcomes for recipients [33], [34] and better predict the functioning of the graft after transplantation.

Finally, some complex issues are highlighted from the behavioural analysis here. Though rotation bias in animals receiving intact pieces of VM tissue continued beyond that seen with dissociated cells (figure 4), the difference between the groups had not (yet) reached statistical significance by 6 weeks post transplantation. This may possibly reflect the maximum effect (on rotation improvement) that half a VM transplant can have on recovering ipsilateral rotation bias in animal models of PD (as the rotation bias of the intact tissue transplant group was only 0.8), or the possibility that pieces of tissue may offer an (albeit delayed) improvement beyond that seen with dissociated cells. Future work in animal models of PD should seek to determine if the trend of improvement in rotation scores in animals receiving intact tissue transplants continues beyond 6 weeks post-transplantation, as rotation scores for this treatment group had not yet levelled off at the end of the present study. It could be the case that intact pieces of tissue are continuing to evolve, and/or grow out of the graft region at 6 weeks post-transplantation, and may offer further improvement beyond such a time course. This seems possible when considering that whole tissue pieces appear to maintain a developmental milieu that embryonic neurons find favourable for survival and growth [26], which may lead to a delay in dopamine cell fibers leaving the graft region and infiltrating the surrounding host tissue. However, there also remains the possibility that whole tissue transplants may themselves suffer some degeneration over time (due to their size, or potential for an increased immune response from the host) and this needs to be explored.

Perhaps more interestingly, however, is the fact that although half a piece of intact E13 VM tissue was able to reduce rotation bias to nearly 0, it did not, on average, reverse the rotation bias (as is seen in many transplantation studies, [35]). This raises the question of whether rotational scores in unilaterally lesioned animal models of PD should return to a null bias (i.e., a rotation score of 0) after transplantation, or whether reversal of rotation is a more appropriate outcome. It seems plausible that a reversed biased of rotation after transplantation in the rodent model of PD may represent over activity of the graft’s function (which is placed unilaterally), and that this might equate to dyskinesias in grafted humans. Future studies looking at longer recovery periods and the recovery of more complex motor tasks in animal models of PD (e.g., stepping, paw reaching, or the corridor task [36], [37]) may help clarify this, and better illustrate how effective small (but more functional) pieces of primary tissue are at recovering motor function in animal models of PD.

### Conclusions

It is hoped that the present work provides some insight into the potential to maximise the effectiveness of small amounts of primary donor tissue for cell replacement of the nigro-striatal system. When the integrity of the tissue was maintained, TH+ cell numbers were increased to approximately double that seen with dissociated cell transplants, and the functioning of the transplants (of pieces of tissue) could be better predicted by the dopamine neuron content within the grafts. In the future, it could be important to directly test the tissue saving potential of grafting whole pieces of tissue by comparing the behavioural recovery of animals receiving dissociated cells or piece of VM tissue in different proportions. If it is found that by transplanting intact pieces of tissue less donor tissue is needed to bring about good motor improvement, then modifying the technique for use in the clinic may be worth exploring. Though stem cells offer greater potential as a source of dopaminergic neurons for cell replacement in PD in the future [7], [38], [39], the time course for generating a stem cell-derived reservoir of neurons for clinical use is uncertain, and it could be the case that maximising the efficacy of the limited supply of primary cells/tissues in the short term is a route worth continuing.
